# Development of mechanically-consistent coarse-grained molecular dynamics model: case study of mechanics of spider silk

**DOI:** 10.1038/s41598-023-46376-6

**Published:** 2023-11-07

**Authors:** S. Momeni Bashusqeh, N. M. Pugno

**Affiliations:** 1 Laboratory for Bioinspired, Bionic, Nano, Meta Materials and Mechanics, University of Trento, Via Mesiano 77, 38123 Trento, Italy; 2 School of Engineering and Materials Science, Queen Mary University of London, Mile End Road, London, E1 4NS UK

**Keywords:** Coarse-grained models, Biomaterials - proteins

## Abstract

Understanding mechanics of spider silk holds immense importance due to its potential to drive innovation in the development of materials with exceptional mechanical characteristics suited for a wide range of applications. Coarse-grained (CG) molecular simulations plays a particularly valuable role in this endeavor, allowing for the efficient investigation of spider silk’s mechanical properties. Our research is centered on the examination of spider silk, which comprises major ampullate silk protein (MaSp1). To achieve this, we developed a CG molecular dynamics model. Our investigation began with a focus on MaSp1 chains subjected to uniaxial tensile load, with comparisons made between the CG model results and all-atom simulations. Subsequently, we extended our simulations to encompass more extensive systems, including fully-ordered MaSp1 bundles undergoing uniaxial static stretching. Through comparison with existing literature, we assess how well the CG model reproduces the mechanical properties of spider silk in highly ordered structures. Furthermore, we explored a scenario where MaSp1 bundles were randomly positioned and stretched, providing valuable insights into silk behavior when the initial structure lacks order. Another simulation involved random positioning, but with some degree of orientation in the loading direction, allowing for a closer examination of the initial structure’s influence.

## Introduction

Spider silk has garnered significant attention due to its remarkable mechanical properties, making it an extraordinary material. What sets it apart is its unique combination of strength, toughness, and elasticity. Spider silk fibers consist primarily of proteins synthesized by specialized glands located in the spider's abdomen. Each type of spider silk showcases distinct mechanical properties. To explore these attributes, numerous studies have employed experimental methods. The findings from these studies have revealed that spider silk fibers possess an exceptional strength-to-weight ratio, surpassing that of any other known material. Various factors, such as temperature, humidity, strain rate, and the silk's protein sequence, can influence its mechanical properties.

Researchers have conducted extensive investigations to examine the impact of humidity on the mechanical properties of spider silk. These studies employ experimental techniques^1–8^ as well as theoretical approaches^9^. The influence of temperature on the mechanical properties of spider silk has been recognized as a critical variable, with several studies emphasizing its significance^5,7,10–12^. Furthermore, the strain rate has been identified as another noteworthy parameter, supported by experimental investigations^3,13–16^, as well as numerical simulation methods like finite element analysis and molecular dynamics simulations^17,18^. Additionally, protein sequence of the silk has been found to be an essential factor impacting its mechanical behavior. Experimental studies^12,13,15,19–25^ and molecular dynamics simulations^26,27^ have been conducted to demonstrate the influence of silk’s protein sequence on its mechanical properties. The design and production of artificial spider silk that can match the strength and extensibility of natural spider silk pose a significant challenge. Researchers are challenged with not only reproducing the molecular structure of silk but also emulating the intricate spinning process that spiders have developed over millions of years. Moreover, they must accomplish this in a manner that is both economically efficient and environmentally sustainable in order to establish the commercial feasibility of artificial spider silk. Meeting challenges such as achieving a high strength-to-weight ratio and striking a balance between strength and extensibility in the synthetic silk are also key considerations in this endeavor. The complexity of the natural material and the numerous factors affecting its mechanical properties contribute to this difficulty. Moreover, additional parameters, such as reeling speed, choice of solvent during production, and post-treatment methods, have been identified as influencing the properties of artificial silk, as demonstrated in previous studies^10,22,28–33^. Studies have revealed that the reeling speed has a beneficial impact on the Young's modulus and tensile strength of the silk, while it has a detrimental effect on the failure strain^10,22,32^. In broad terms, the production of fibers typically involves two primary techniques: wet spinning and dry spinning. In the spinning process of spider silk, various solvents can be used to dissolve and extrude the silk proteins into fibers. Some of the solvents employed in previous works include water, ethanol and methanol^28,29^. Different solvents can yield variations in the properties of the resulting silk fibers, and the choice of solvent can impact mechanical properties like fiber tensile strength, extensibility, and Young’s modulus. Different approaches can be considered for post-treating the spun silks. For instance, it has been demonstrated that subjecting synthetic silk to methanol or ethanol vapor during post-processing^30^ or addition of acetonitrile and polyethylene glycol to the collection bath^31^ can increase the β-sheet content, thereby enhancing the material's mechanical properties. In another effort to enhance the mechanical properties of synthetic spider silks^33^, Glutaraldehyde was introduced into the spinning solution, followed by stretching the spun silk in an alcohol-based stretching bath.

However, continuous research on the properties and behavior of spider silk holds promise in providing valuable insights and knowledge that may eventually facilitate the development of synthetic spider silk with comparable properties.

Although experimental approaches have been the primary means of studying the mechanics of spider silk, they have inherent limitations, much like any other method. These limitations include the considerable cost and resource demands associated with experimentation, time-consuming procedures, restricted control over experimental conditions, particularly in intricate biological systems, invasive techniques that may perturb the system, restricted spatial resolution hindering the observation of detailed molecular interactions, and susceptibility to variations in conditions, equipment, or human error, all of which can impact the reproducibility and dependability of results.

In contrast, computational tools offer particular value in investigating the behavior of the material at the nanoscale. Despite their potential, only a limited number of studies have utilized computational methods, such as the molecular dynamics approach, to explore the mechanics of spider silk. The scarcity of such computational studies can be attributed to the significant computational costs linked to all-atom molecular dynamics simulations, along with the inherent complexity of the molecular structure of spider silk. In all-atom molecular dynamics simulations, it is imperative to consider each individual atom. This methodology necessitates the incorporation of interactions among all atoms, resulting in a substantial computational cost during each time-step of the simulation. Consequently, the utilization of the all-atom molecular dynamics method is commonly limited to smaller molecular systems. To address the challenges associated with the all-atom molecular dynamics method, it becomes necessary to reduce the number of degrees of freedom, consequently decreasing the computational workload. Coarse-grained (CG) molecular dynamics models achieve this by grouping multiple atoms into a single interacting bead, substantially diminishing the computational requirements for conducting the simulations.

Although there are number of CG molecular dynamics models available for simulating proteins^34^, in addition to the bead-spring model developed for spider silk^27,35,36^, it is important to note that, to the best of the authors' knowledge, no single model currently exists that accurately reproduces the mechanical properties of spider silk. The limitation with CG models is that they have not been specifically optimized to replicate the mechanical behavior of the system under investigation. On the other hand, while the bead-spring model is relatively simple, its applicability may be questioned in scenarios involving different loading conditions, as it is primarily developed based on a specific load–displacement curve derived from all-atom molecular dynamics simulations. Therefore, the development of a coarse-grained molecular model which is simple and capable of capturing the behavior of spider silk under mechanical loading is considered a crucial and ongoing research endeavor in this field.

The primary objective of this study is to create a precise coarse-grained molecular dynamics model capable of simulating the mechanical behavior of spider silk composed of MaSp1 proteins under various load conditions. To evaluate the accuracy and reliability of the coarse-grained molecular model, its results are compared to those obtained from all-atom simulations and previously reported findings in the literature. This comparison serves as a crucial step in assessing the validity of the model by verifying its ability to reproduce important characteristics and behaviors observed in both the all-atom simulations and the existing literature. By demonstrating consistency and agreement with established results, the model's validity is established, and its capability to capture the essential features of spider silk's mechanical behavior is confirmed.

The methodology employed in this study is the most significant aspect, as it enables the construction of CG molecular dynamics models for spider silk using any desired gene sequence. The approach presented here serves as a framework that can be utilized to design and optimize silk proteins beyond the specific gene sequence examined in this research. This adaptability creates exciting possibilities for silk protein engineering and contributes to the overall progress of biological materials design and development.

The authors express their aspiration to establish a solid foundation for future computational investigations that focus on unraveling the extraordinary properties of spider silk. Through their work, they aim to enhance the understanding of spider silk's mechanical behavior, thereby paving the way for new avenues of exploration and advancement in this field.

## Results

In this section, our focus is on investigating the response of spider silk to uniaxial static stretching using the developed CG model. We subject the system to incremental uniaxial stretching, where each increment corresponds to a strain of 0.05%. By gradually applying the stretching, we can observe and analyze the behavior of the silk as it undergoes deformation. Mechanical loading can be applied at various strain rates, but it's essential to recognize a limitation associated with this aspect. Due to computational costs associated with MD simulations, it’s almost impossible to conduct the simulations at a reasonable strain rate. Typically, the strain rates considered in the literature for molecular dynamics (MD) simulations fall within the range of 10^7^–10^8^ 1/s which is unrealistic. While we theoretically could perform simulations at such high strain rates, there would be a lack of suitable reference experimental data for comparison, making the results less meaningful and challenging to interpret effectively. In light of these considerations, we opted to apply mechanical loading statically. This choice was motivated by the availability of published experimental data that we could use for direct comparison with our results. After each stretching increment, the system is equilibrated to allow for relaxation. During this equilibration, the system is maintained at a constant temperature of 300 K and subjected to lateral pressure components of 1 atm. While it is possible to conduct the simulations at different temperatures, our primary focus is examining mechanical behavior of the silk under ambient conditions. This equilibration period, lasting for 200 picoseconds followed by energy minimization, allows the system to reach a stable and relaxed state following the applied strain. Through this process, we can study the response of the spider silk to pure static stretching. In the subsequent sections, we present the results obtained from these simulations, discussing the mechanical response of spider silk under uniaxial loading.

### Fully- ordered spider silk

**Figure 1:**
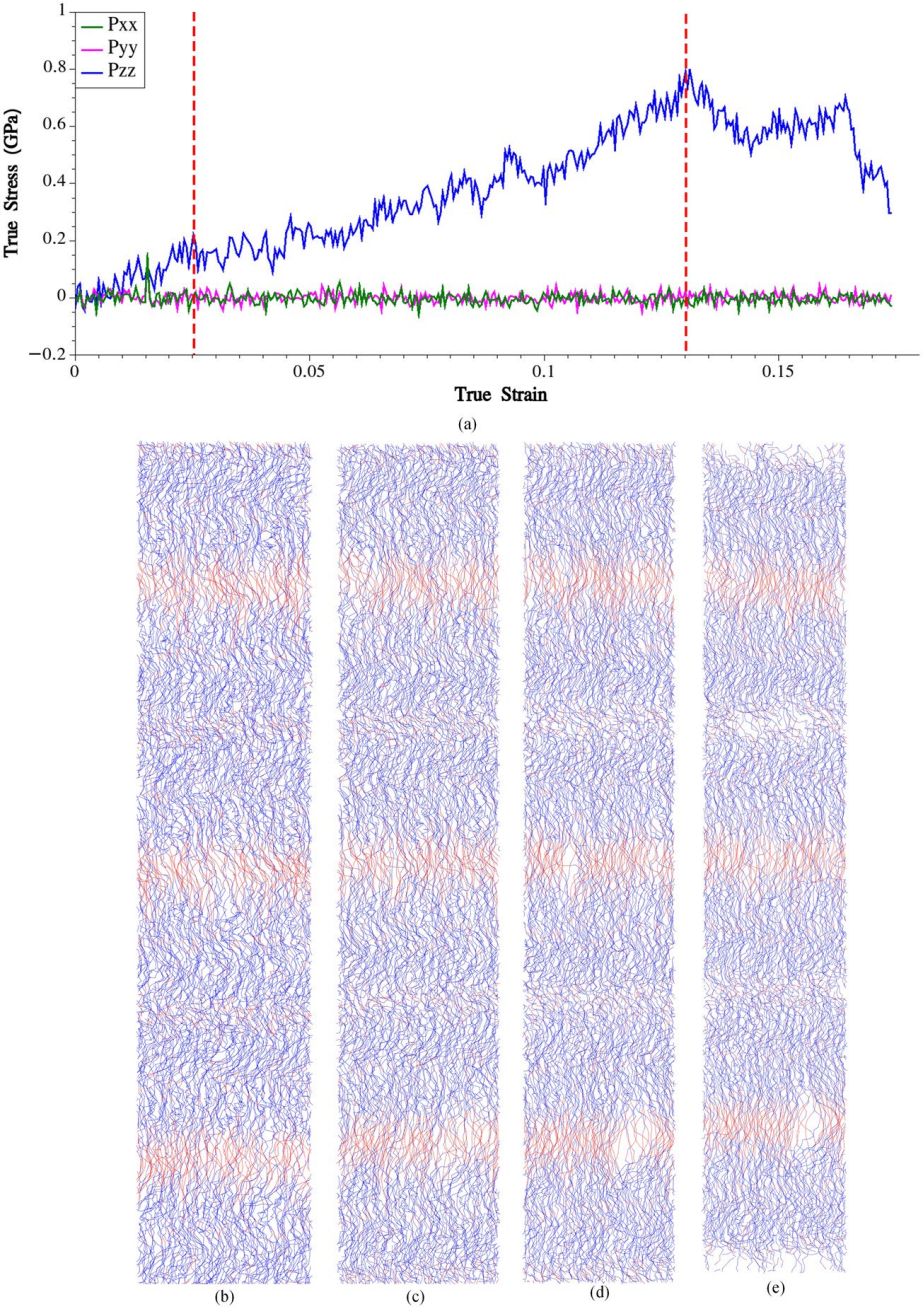
(**a**) Stress–strain curve obtained from CG simulation of the fully-ordered system. Fully-order silk at (**b**) ε = 0 (**c**) ε = 0.06 (**d**) ε = 0.13 (**e**) ε = 0.18.

In this section, we investigate the mechanical response of spider silk in a configuration where all the chains are oriented along the loading direction. To ensure equilibration in a reasonably large system, we replicate the smaller equilibrated system (see section “Methods”) 3 times in the X direction, 2 times in the Y direction, and 3 times in the Z direction. The loading is applied in the Z direction. The system is then allowed to equilibrate at a temperature of 300 K and a pressure of 1 atm for a duration of 30 ns. During the last 1 ns of the equilibration process, the average density and longitudinal length of the simulation box remain constant at 1.4 g/cm^3^ and 394.17 Å. The obtained density of 1.4 g/cm^3^ from the equilibrated system using the developed CG model is indeed in good agreement with the reported density of 1.35 g/cm^3^ for silk from *Nephila Clavipes* spider in literature, which is primarily composed of MaSp1 and MaSp2 proteins in a ratio of approximately 4:1^37^. This agreement indicates that the CG model successfully captures the overall packing and arrangement of the spider silk molecules, leading to a density value that aligns well with experimental observations. Noteworthy is that in the previous section where we simulated only one bundle of MaSp1 chains, the average density and longitudinal length of the simulation box during the last 1 ns of the equilibration were obtained as 1.28 g/cm^3^ and 114.6 Å from CG simulations (1.25 g/cm^3^ and 110.2 Å from all atom simulations). The observation that the density and longitudinal length of the simulation box increased when simulating multiple bundles of MaSp1 chains compared to a single bundle is indeed significant. The increase in both density and longitudinal length (per bundle) indicates that simulating the system at a larger scale, with multiple bundles, better reproduces the structural properties of spider silk in line with experimental evidence. By simulating multiple bundles, the interactions between the chains are more accurately represented, allowing for a more realistic packing and arrangement of the silk molecules. This improved representation of intermolecular interactions leads to a closer match between the simulated structural properties and experimental observations. Following the loading procedure outlined earlier, the obtained stress–strain curve is presented in Fig. 1.

The stress–strain curve depicted in the figure reveals three distinct regimes. In the first regime, characterized by linear behavior, the longitudinal stress (Pzz) exhibits a consistent increase with applied strain, indicating the elastic response of the fully-ordered silk. The yield point is observed at a true strain of 0.025, corresponding to a yield stress of approximately 0.20 GPa. By fitting a linear curve to the elastic regime, the initial Young modulus is determined to be 8.04 GPa. On the other hand, failure of the fully-ordered silk is observed at a true strain of 0.13, with a corresponding failure stress of 0.79 GPa. Another aspect to note is that the silk tends to fail in areas where the bundles become entangled.

These results were compared with experimental findings reported earlier^14^, where major ampullate silk from the orb-weaving spider *Nephila Clavipes* was tested under low and high strain rates. Since our simulation focused on pure static loading of spider silk, we compared the obtained results with the findings reported for low strain rate conditions (0.001 s^-1^). The initial Young modulus was reported as 12.06 ± 1.61 GPa, which differs notably from the initial Young modulus obtained in our work. However, it is important to note that despite the discrepancy in the initial Young modulus, the prediction of silk failure in our work matches very well. The failure strain and stress reported were 0.12 ± 0.022 and 0.83 ± 0.08 GPa, respectively, which closely align with the failure strain and stress observed in our simulation. We would also like to highlight the findings from another experimental work^33^, where the initial Young modulus and failure stress of silk from the *Nephila Clavipes* spider were reported as 8.4 GPa and 1.73 GPa, respectively. It is important to note that the silk was subjected to a high strain rate of 0.1 s^-1^ and was collected from the spider's web, which is in contrast to the reeling condition of 200 mm/s in the other experimental work^14^. The modulus of toughness holds significant importance within this context. To determine this parameter, we calculated the area under the stress–strain curve until the point of failure strain. Our calculations yielded a value of 44.9 MJ/m^3^ for the modulus of toughness. It is crucial to highlight that modulus of toughness of the silk was reported to be 63.16 ± 16.30 MJ/m^3^ under low strain rate condition^14^ which is noticeably lower than the findings presented in the other reference^33^ (206 MJ/m^3^). However, it is important to note that the modulus of toughness obtained in our study aligns closely with the minimum modulus of toughness reported^14^. Indeed, considering the various uncertainties and factors that can influence the mechanical behavior of spider silk, including those mentioned earlier, it is important to acknowledge the limitations and potential variations in experimental results; this fact is pronounced when we see from the literature that diet of the spider could also be effective on the mechanics of spider silk^38^.

Nevertheless, despite the challenges faced, the ability of the developed CG model to qualitatively replicate the mechanical behavior of spider silk with reasonable accuracy is a noteworthy accomplishment for several reasons. Firstly, it offers a means to delve into the underlying mechanisms and structural characteristics that influence the material's mechanical properties. Secondly, it enables the exploration of various scenarios and configurations that might be challenging or time-consuming to investigate through experimental means alone. Lastly, it establishes a platform for future investigations and studies that can advance our comprehension of spider silk, while also guiding the design of biomimetic materials possessing desired gene sequences and mechanical properties.

### Semi- ordered and fully-random spider silk

**Figure 2:**
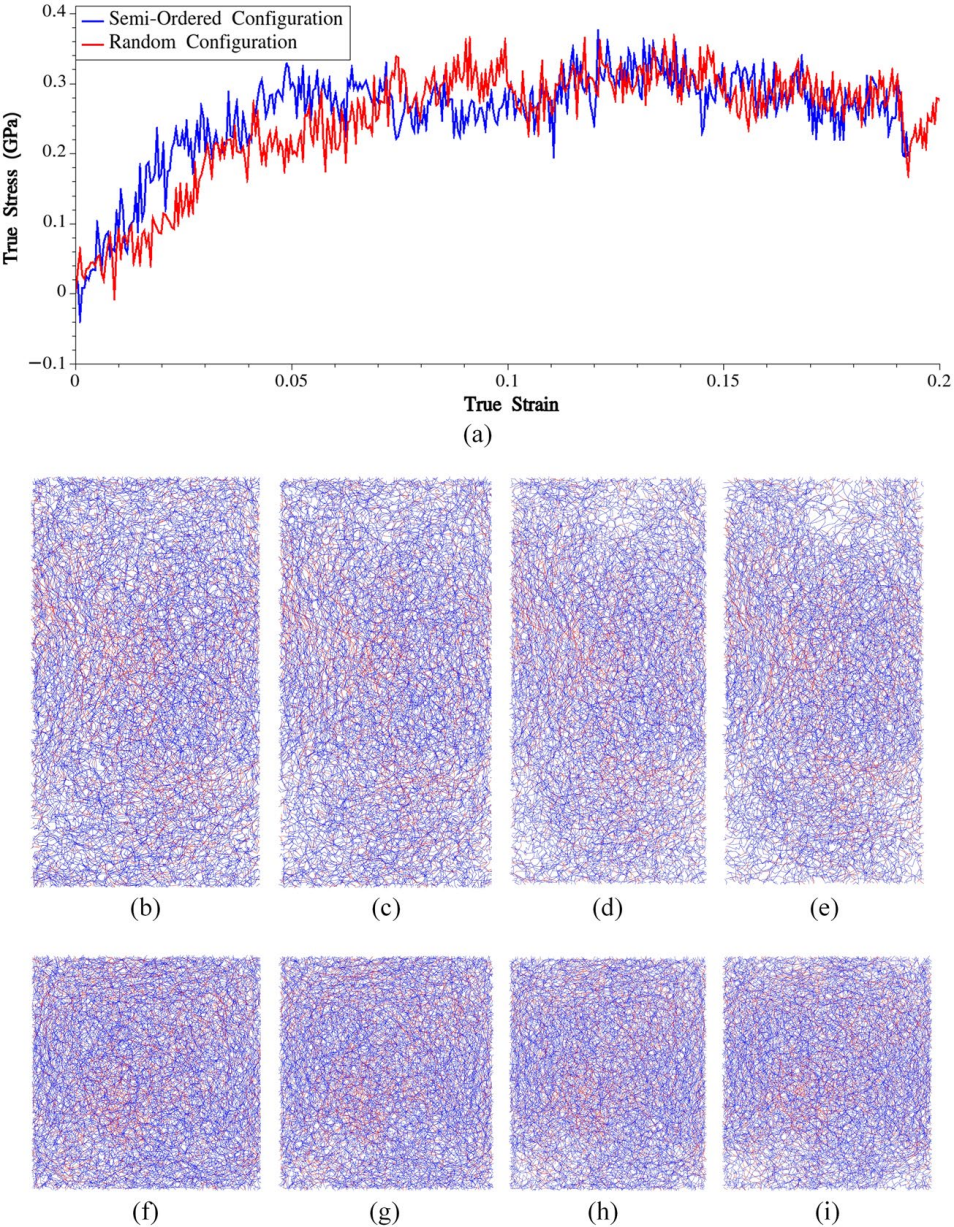
(**a**) Stress–strain curves obtained from CG simulations of the fully-random and semi-ordered systems. Semi-order silk at (**b**) ε = 0 (**c**) ε = 0.06 (**d**) ε = 0.13 (**e**) ε = 0.18. Fully-random silk at (**b**) ε = 0 (**c**) ε = 0.06 (**d**) ε = 0.13 (**e**) ε = 0.18.

In this section, we aim to study the influence of MaSp1 chain orientation on the mechanical behavior of the material. By varying the arrangement and alignment of the chains, we can analyze how different orientations affect the material's response under specific loading conditions. This investigation can provide valuable insights into the role of chain orientation in determining the mechanical properties of spider silk, contributing to a deeper understanding of the structure–property relationships. To explore this, we have created two different initial configurations. In the first configuration, we randomly placed 20 copies of the equilibrated system obtained from section "Conclusion" within a simulation box with dimensions of 126.5 Å × 126.5 Å × 126.5 Å. The random placement of these copies was performed using Moltemplate^39^ and Packmol^40^. This configuration allows for a fully random arrangement of the MaSp1 chains, without any specific alignment or orientation. In the second configuration, we placed 20 copies of the equilibrated system within a simulation box with dimensions of 98.5 Å × 98.5 Å × 197 Å. The choice of these dimensions was made to impose a certain degree of orientation in the molecular system while maintaining a partially random configuration. Upon examining the coordinates of the beads in the constructed initial configuration, it can be observed that 13 out of 20 bundles (or 65%) were perfectly aligned in the Z direction. This configuration strikes a balance between oriented and random molecular arrangements, enabling the investigation of the material's mechanical response under conditions where there is some level of orientation present, but not a complete alignment or complete randomness. After equilibrating the two molecular systems at ambient conditions, they were subjected to uniaxial stretching, similar to the fully-ordered system. In Fig. 2, the obtained stress–strain curves for these two systems and snapshots depicting the two systems at different stages of loadings are presented.

The semi-ordered system initially exhibits higher stiffness but fails to surpass the maximum stress achieved in the fully random system, this is indeed interesting. It suggests that the specific arrangement and alignment of the chains in the semi-ordered configuration may enhance the initial stiffness of the material, but it does not necessarily translate into a higher ultimate strength. The reason why the stress component in the loading direction cannot significantly exceed the yield stress can be attributed to the fact that in these systems, the applied incremental strain manifests partly as separation between the chains that are not aligned in the loading direction. The separations between the chains are governed by Lennard–Jones potential, which constitutes the only non-bonded interaction within the system. It's crucial to emphasize that Lennard–Jones interaction is characterized by its short-range nature, signifying that the force arising from this interaction decreases rapidly as the chains move further apart. However, this scenario differs in the fully-ordered systems, where all the chains are aligned in the loading direction, effectively withstanding the applied load due to the combined effects of bonding interactions and non-bonded interactions (where chains are intricately entangled; the bundle was replicated 3 times in loading direction).

The observation that the stress component in the loading direction remains relatively constant around 0.2 GPa (within a specific strain range) in the fully random and semi-ordered systems is interesting. This suggests that there might be a common threshold or limit in the material's response, regardless of the initial chain orientation or arrangement. It is important to note that the yield strength obtained from the fully-ordered system was also 0.2 GPa. This indicates that the fully random and semi-ordered systems reach a similar stress level as the fully-ordered system before undergoing significant deformation.

## Conclusion

In this study, we developed a novel coarse-grained molecular dynamics model to explore the mechanics of spider silk composed of MaSp1 proteins. To validate the accuracy and consistency of our CG model, we performed simulations on a simple molecular system consisting of 16 MaSp1 chains, comparing the results with those obtained from the all-atom model. Subsequently, we expanded our investigations to a larger system comprising MaSp1 bundles aligned in the loading direction and subjected to uniaxial static stretching. By comparing our findings with existing literature, we successfully demonstrated the effectiveness of our model in reproducing crucial properties of spider silk. Furthermore, we examined molecular systems consisting of randomly positioned MaSp1 bundles under mechanical loading. Our results highlighted that any misalignment of the bundles with respect to the loading direction can significantly reduce the tensile strength of the silk. Additionally, we revealed that regardless of the orientation of the bundles, there exists a specific stress level beyond which the behavior of the silk undergoes a notable change.

## Methods

In our endeavor to develop the CG model, we adopted a methodology that involves the calibration of CG potentials to ensure energy conservation between the CG and all-atom molecular models. This approach allowed us to create a CG model specifically for MaSp1 protein, enabling simulations of silk at larger scales. The force field of the CG model comprises two types of potential functions: bonded and non-bonded interactions. The overall potential energy (E_total_) of the system is expressed as the sum of different energy terms associated with changes in bond length (E_b_), bond angle (E_a_), dihedral angle (E_d_), van der Waals (vdW) interactions (E_vdW_), and the constant free energy of the system (U_o_), represented as follows:

1 $${E}_{total}={E}_{b}+{E}_{a}+{E}_{d}+{E}_{vdW}+{U}_{o}$$

To prioritize the simplicity of the model, we have represented the mathematical formulas for the individual energy terms that contribute to a single interaction as follows:

2a $${E}_{b}={K}_{b}{\left(r-{r}_{o}\right)}^{2}$$

2b $${E}_{a}={K}_{a}{\left(\theta -{\theta }_{o}\right)}^{2}$$

2c $${E}_{d}={K}_{d}{\left(\varphi -{\varphi }_{o}\right)}^{2}$$

2d $${E}_{vdW}=4\varepsilon \left[{\left(\frac{\sigma }{r}\right)}^{12}-{\left(\frac{\sigma }{r}\right)}^{6}\right]$$

where ${K}_{b}$, ${K}_{a}$ and ${K}_{d}$ stand for the stretching, bending and torsional stiffness, respectively. Also, ${r}_{o}$, ${\theta }_{o}$ and ${\varphi }_{o}$ represent equilibrium bonding length, bending angle and torsional angle, respectively. For non-bonded interaction, $\varepsilon$ and $\sigma$ are interaction strength and zero-crossing distance for the potential, respectively. In the following sections, we calibrate the parameters for the above functional forms. To carry out this calibration, we employ verification processes that involve all-atom molecular simulations, utilizing CHARMM27 force field^41^ to describe molecular interactions where LAMMPS^42^ is used to do both all-atom and CG simulations. The non-bonded interactions are modeled using vdW and Coulombic energy terms, with a potential cutoff of 1.2 nm being used for the calculations.

### Coarse-grained stretching potentials

The initial step in the coarse-graining process involves mapping a group of atoms from a full atomistic system onto a single CG bead. In this study, a CG model for MaSp1 protein was developed using a one-bead-per-amino-acid residue approach (see Supplementary Fig. S1). The center of the CG bead was positioned at the center of mass for the corresponding residue, while pseudo-atoms with atomic masses equal to that of the respective residue were defined. The amino acid sequence of the MaSp1 protein (in one-letter-amino acid codes) is GGAGQGGYGGLGSQGAGRGGLGGQGAG/AAAAAA/GGAGQGGYGGLGSQGAGRGGLGGQGAG. With the implementation of this CG model, a significant reduction of 10.6-fold in the number of degrees of freedom (DOF) for the system was achieved compared to a full atomistic system. This reduction in DOF enables more computationally efficient simulations while still capturing the essential behavior and characteristics of the system. In the CG model, the residues are numbered from left to right, resulting in residue No. 1 to No. 60.

**Figure 3:**
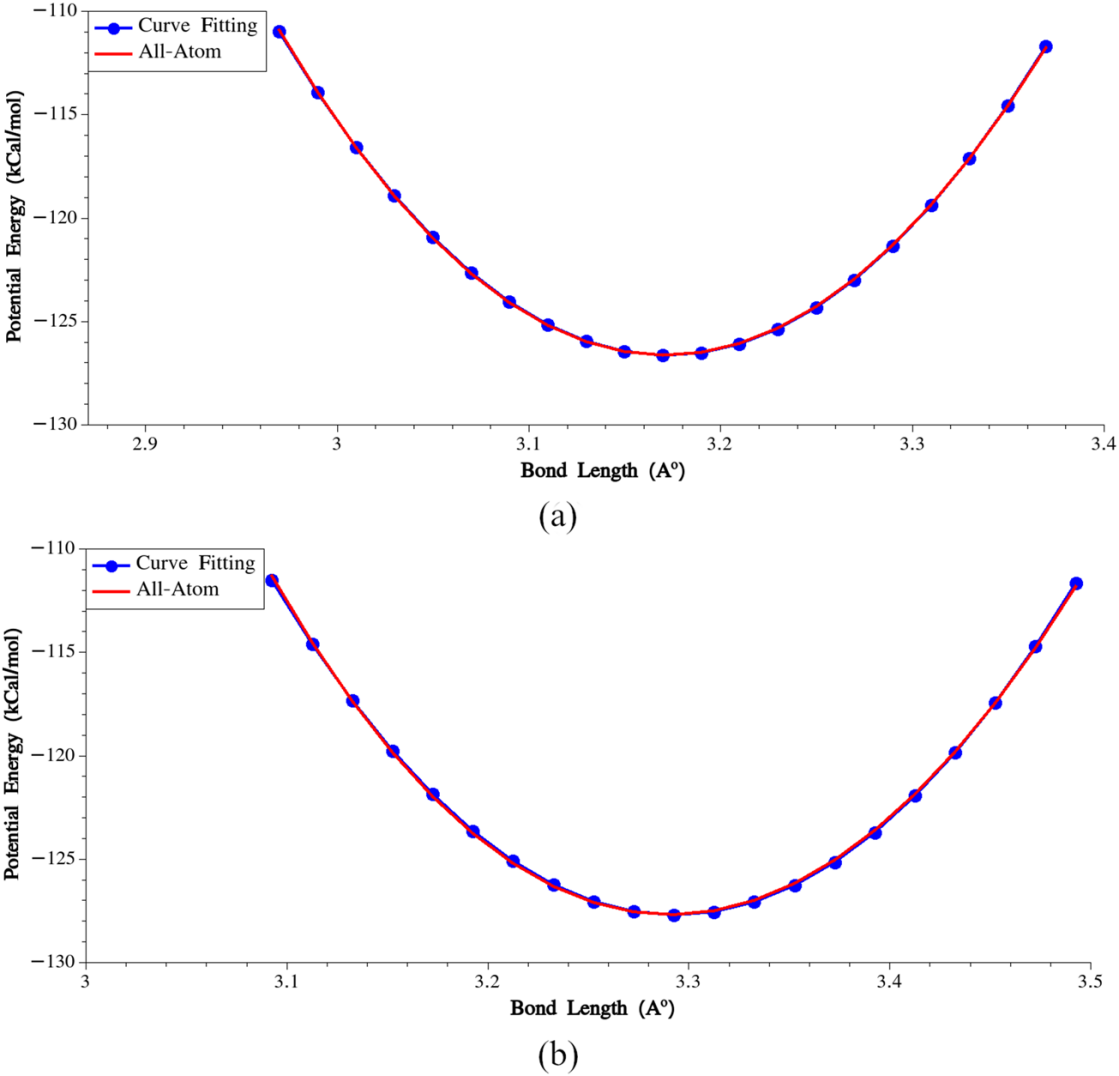
(**a**) Calibration of bonding potential for the bond connecting residues No. 9 and 10, (**b**) Calibration of bonding potential for the bond connecting residues No. 30 and 31.

To calculate the stretching potential between residues No. (i) and (i + 1), the following steps were undertaken:

(i) The potential energy of a single MaSp1 chain was minimized using the Hessian-free truncated Newton algorithm (in all-atom model).
(ii) All the residues except No. (i) and (i + 1) were deleted.
(iii) Residue No. (i + 1) was incrementally displaced along the connecting line between the centers of the two residues.
(iv) At each increment, the total potential energy of the system was recorded and plotted.
(v) We identified the bond length at which the minimum energy occurs, and then fitted a harmonic potential to the energy variation in the vicinity of the bond length corresponding to the lowest energy. In Fig. 3, variations of potential energies and the fitted harmonic potentials are plotted for G-G (residues No. 9 and 10) and A-A (residues No. 30 and 31) pairs.

Through these steps, the stretching stiffness and equilibrium bond length were determined for all pairs of connecting beads. The calculated parameters are presented in Supplementary Table S1. It is important to note that although there are similar pairs of connected beads in the model, we treated them as distinct entities. In Supplementary Table S1, it can be observed that the potential parameters obtained for the bonds between G–Y and Y–G pairs, as well as for G–R and R–G pairs, differ from each other. This distinction arises because non-bonded interactions were considered in the calculation of the potential energy, while these interactions between connected beads are not considered in subsequent CG simulations.

### Coarse-grained bending potentials

Similar to the previous section, the following steps were taken to calculate the bending potentials associated with each bending triple:

(i) The potential energy of a single MaSp1 chain was minimized.
(ii) All the other residues except for the three in the bending triple (residues No. i, i + 1 and i + 2) were deleted.
(iii) Residues No. (i) and (i + 1) were kept fixed, while residue No. (i + 2) was incrementally rotated around residue No. (i + 1) in the plane formed by the triple.
(iv) The potential energy was calculated at each increment, allowing the bending potential associated with the bending triple to be calculated. When calculating the potential energy, we did not take into account the energy associated with non-bonded interactions.
(v) The minimum of the energy variation was found, and a harmonic potential was fitted to the energy variation in the vicinity of the minimum energy configuration.

**Figure 4:**
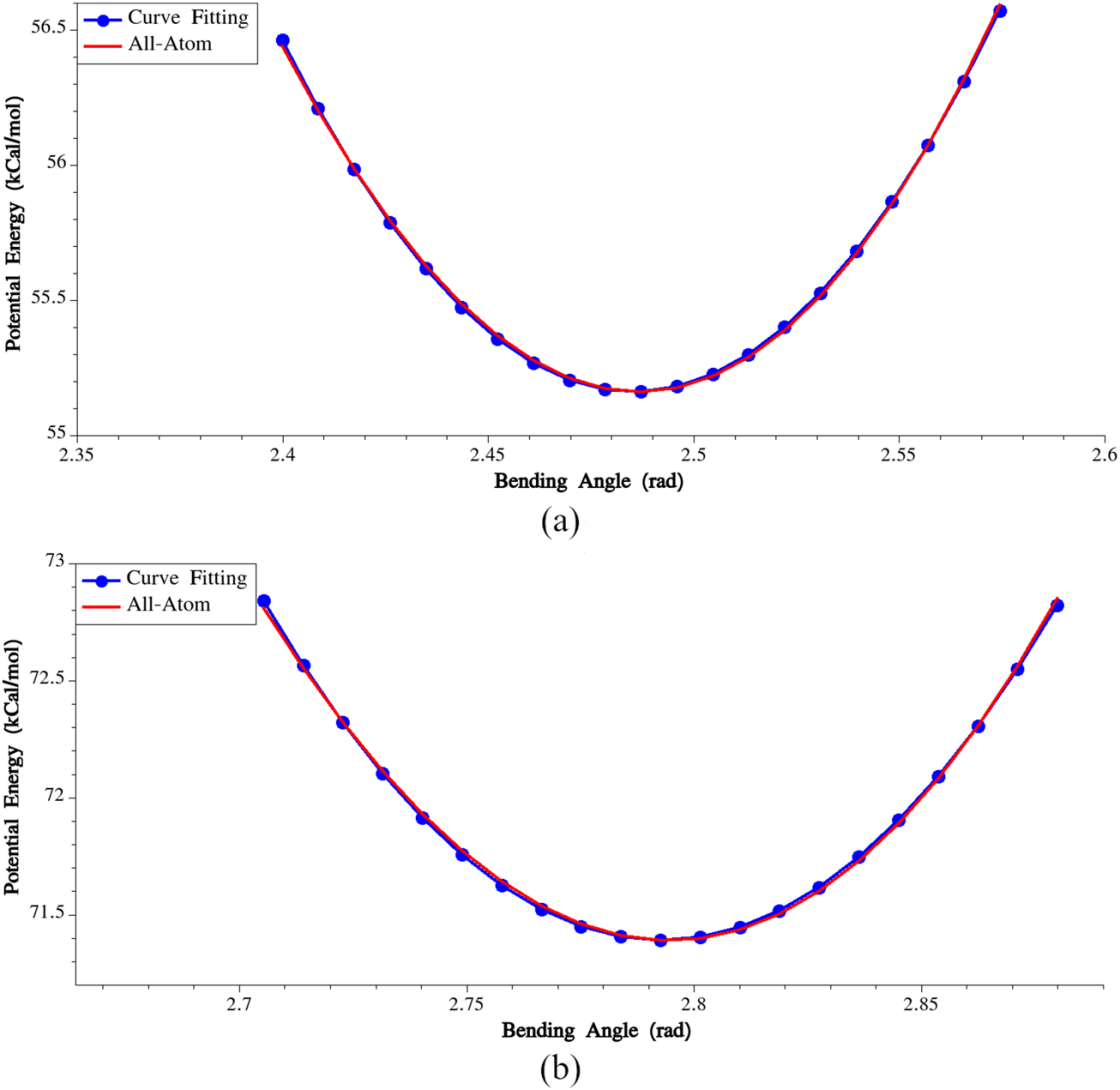
(**a**) Calibration of bending potential for the angle formed by residues No. 2, 3 and 4, (**b**) Calibration of bending potential for the angle formed by residues No. 29, 30 and 31.

In Fig. 4, variations of potential energies and the fitted harmonic potentials are plotted for G–A–G (residues No. 2, 3 and 4) and A–A–A (residues No. 29, 30 and 31) triples. By following these steps, the bending stiffness and the equilibrium bending angle could be calculated for all bending triples, as presented in Supplementary Table S2.

The calculated bending stiffness for G–Y–G and G–L–G triples is noticeably higher compared to the stiffness obtained for the other triples. This significant difference in stiffness emphasizes the distinct mechanical behavior of these particular triplets. Additionally, an interesting observation is the equilibrium bending angles in the poly-Alanine region (residues No. 28–No. 33), which measure approximately 162 degrees. This finding underscores the unique structural characteristics of this specific region within the protein sequence.

### Coarse-grained torsion potentials

**Figure 5:**
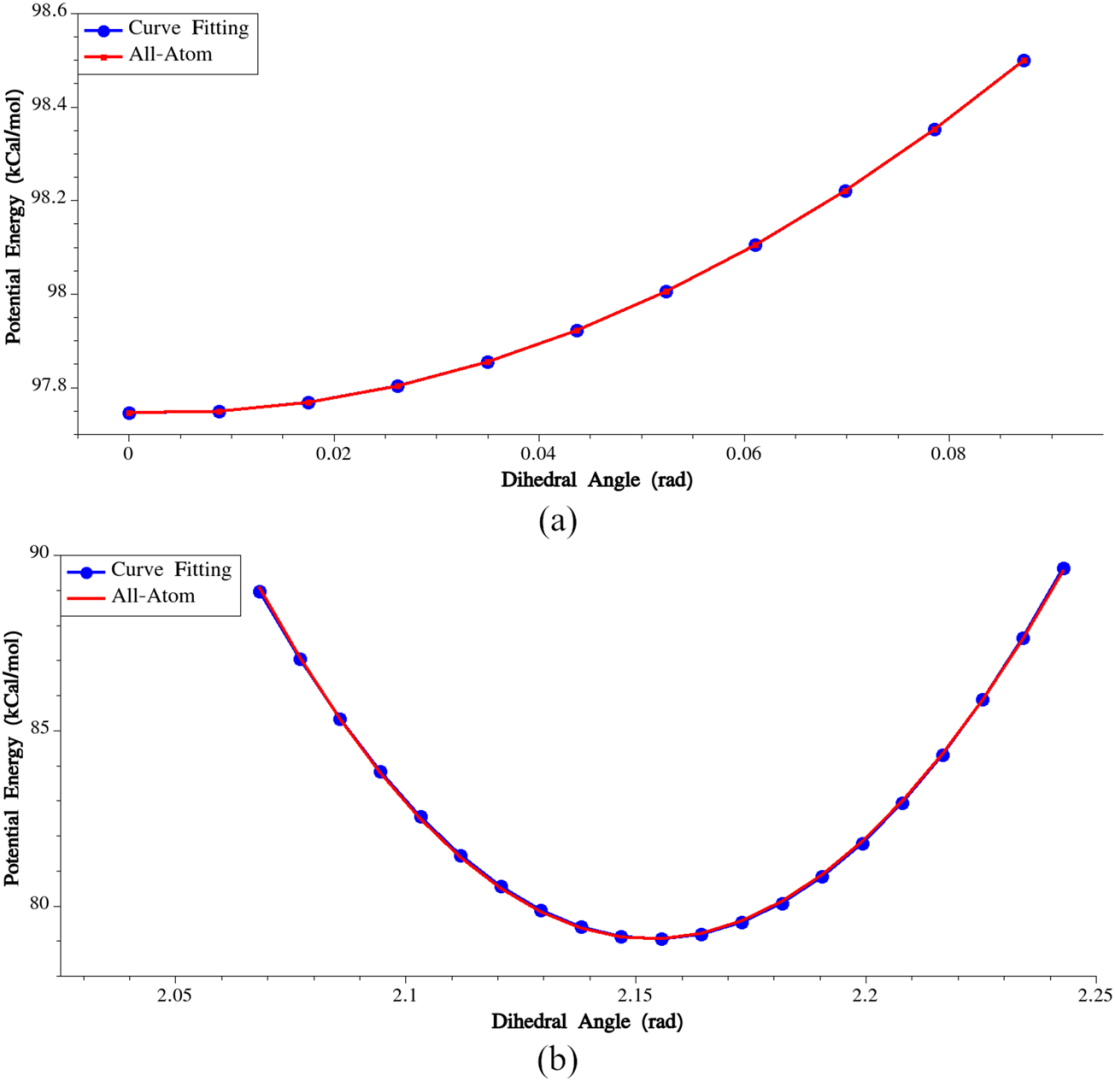
(**a**) Calibration of dihedral potential for the dihedral angle formed by residues No. 3, 4, 5 and 6, (**b**) Calibration of dihedral potential for the dihedral angle formed by residues No. 29, 30, 31 and 32.

In this section, coarse-grained torsion potentials are computed for each four consecutive residues. To calculate the unknown constants, the following steps were followed:

(i) The potential energy of a single MaSp1 chain was minimized.
(ii) All the other residues except for the four in the torsion (residues No. i, i + 1, i + 2 and i + 3) were deleted.
(iii) Residues No. (i), (i + 1) and (i + 2) were kept fixed, while residue No. (i + 3) was incrementally rotated around the connecting line between residues No. (i + 1) and (i + 2). Its projection on the line was considered as the center of rotation. By doing so, we could ensure that the energy variation is solely associated with torsion.
(iv) The total potential energy of the system was recorded and plotted at each increment. Notably, non-bonded interactions between nonadjacent residues were not taken into account in the calculation of the energy variation.
(v) The minimum of the energy variation was found, and a quadratic potential was fitted to the energy variation in the vicinity of the minimum energy configuration. In Fig. 5, variations of potential energies and the fitted harmonic potentials are plotted for A–G–Q–G (residues No. 3, 4, 5 and 6) and A–A–A–A (residues No. 29, 30, 31 and 32) residues. The obtained torsion potentials are provided in Supplementary Table S3.

Similar to the previous section where certain triples exhibited significantly higher bending stiffness, we have also identified instances where the torsional stiffness is notably higher than the average stiffness calculated. These cases indicate the presence of specific conformations or structural motifs that contribute to the increased torsional resistance in those regions.

### Non-bonded vdW potentials

**Figure 6:**
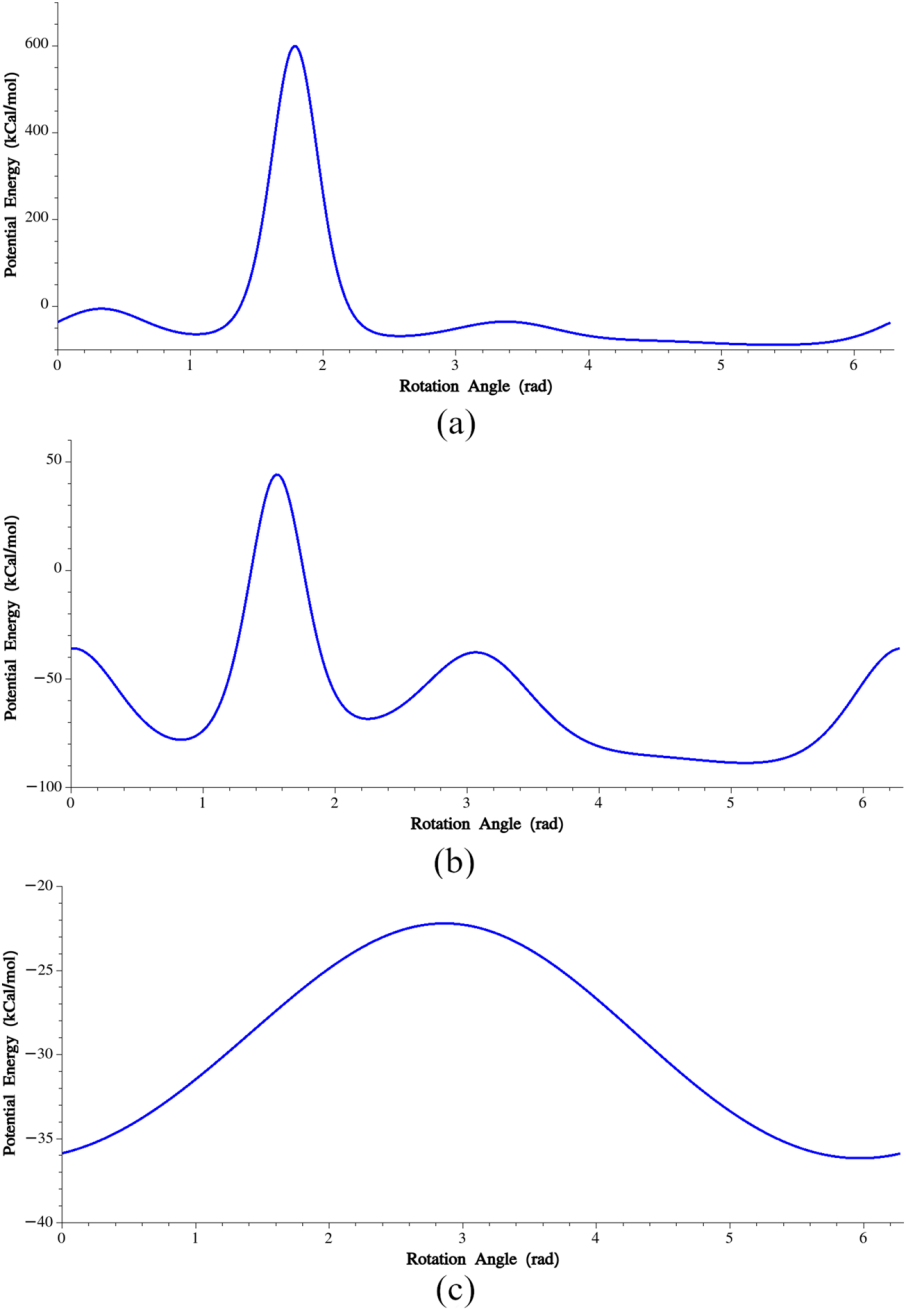
(**a**) Potential energy vs. rotation around X, (**b**) Potential energy vs. rotation around Y, (**c**) Potential energy vs. rotation around Z.

The calibration of non-bonded van der Waals (vdW) interactions was specifically performed on similar beads, while a geometric mixing rule was applied to calculate interactions between non-similar beads. To carry out this calibration, two similar beads (referred to as beads I and J) were positioned at a specific distance, and then bead J was displaced along the connecting line. During this displacement, the total potential energy of the system was recorded. It is important to note that the energy profile during this displacement is influenced by the orientations of the residues, primarily due to electrostatic interactions. This dependency on residue orientations is clearly demonstrated in Fig. 6, which depicts the variation of potential energy as bead J (in a G-G non-bonded pair) is rotated around the X, Y, and Z axes while maintaining the positions of both beads' centers of masses.

Considering the dependency of the potential energy on the orientations of the residues, we recorded the rotation angles at which the minimum potential energy occurred (TetaX, TetaY, and TetaZ). Subsequently, we considered three scenarios to calibrate the non-bonded interactions:

- The bead J was rotated around the X axis equal to TetaX and displaced along the connecting line between beads I and J.
- The bead J was rotated around the Y axis equal to TetaY and displaced along the connecting line between beads I and J.
- The bead J was rotated around the Z axis equal to TetaZ and displaced along the connecting line between beads I and J.

**Figure 7:**
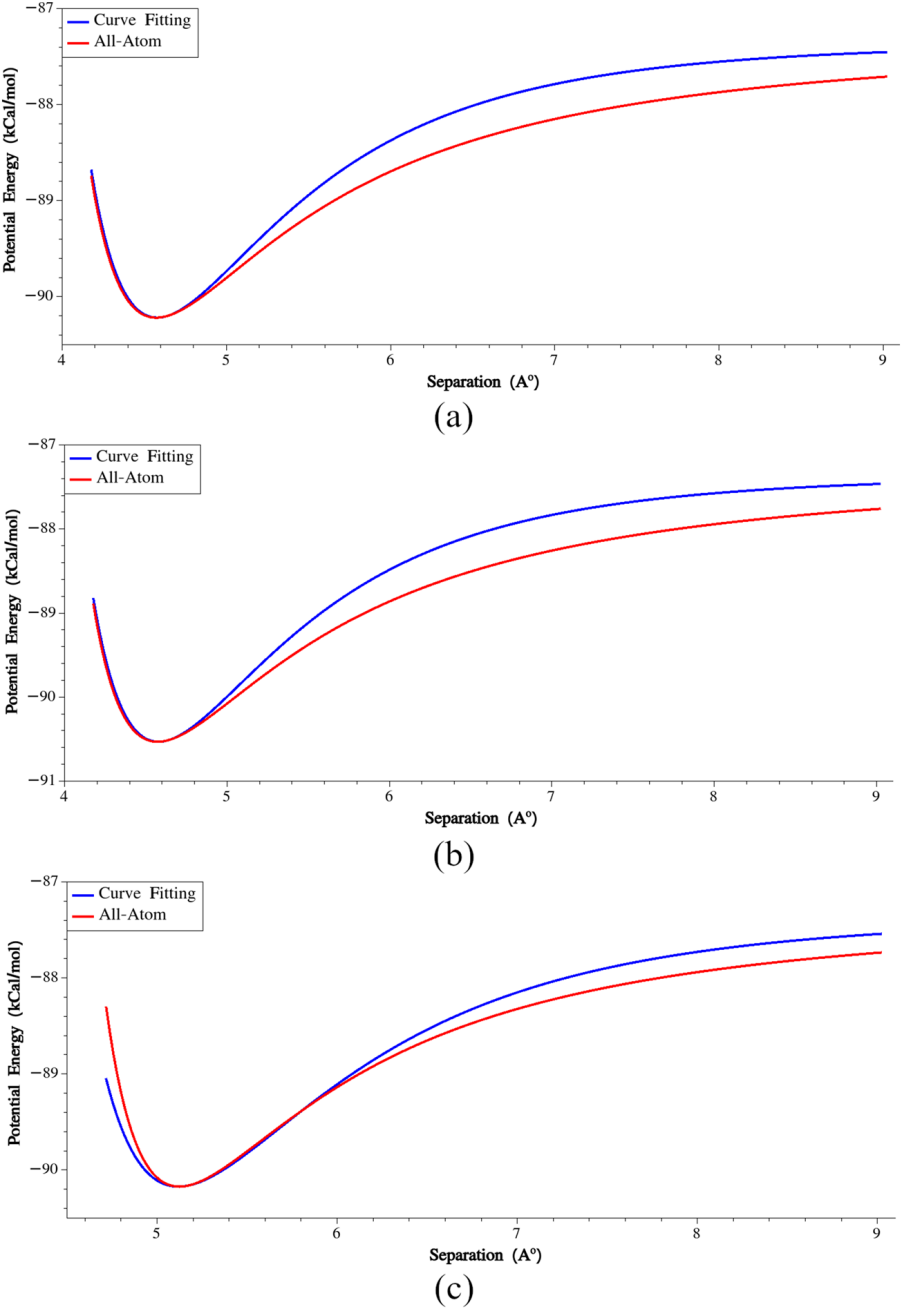
(**a**) Potential energy vs. separation (the bead J was rotated around the X axis), (**b**) Potential energy vs. separation (the bead J was rotated around the Y axis), (**c**) Potential energy vs. separation (the bead J was rotated around the Z axis).

For each scenario, we analyzed the variation of potential energy with separation and fitted a Lennard–Jones (LJ) potential function to the region around the minimum energy configuration. This is illustrated in Fig. 7 for the G–G non-bonded pair. By performing these fits, we obtained LJ parameters for each scenario. To determine the calibrated parameters, we took the averages of the obtained parameters across the different scenarios. These calibrated parameters serve as the representative values for the non-bonded interactions in the coarse-grained model (Supplementary Table S4).

### Verification of the developed CG model

In this section, a fully periodic system comprising 16 MaSp1 chains is subjected to equilibration and static stretching using both all-atom and CG molecular dynamics simulations. To set up the initial configuration of the system, the chains are arranged in a 4 × 4 lattice. This structure incorporates anti-parallel stacking in the hydrogen bonding direction and parallel stacking in the side-chain direction (see Supplementary Fig. S2). Following the initial configuration setup, both the all-atom and CG systems underwent an equilibration process. The systems were initially heated to 800 K and equilibrated for 10 ns. Subsequently, they were cooled down to 300 K over a duration of 10 ns under the NVT ensemble. The equilibration continued at 300 K and 1 atm for an additional 30 ns, during which the pressure components were independently controlled. The convergence of the system's total energy and box dimensions indicated that the obtained structures had reached an equilibrium state.

In the simulations, the Nose–Hoover thermostat and barostat were employed to regulate the temperature and pressure components, respectively. It is important to note that different timestep strategies were used for the all-atom and CG simulations. In the all-atom simulations, a timestep of 1 fs was utilized to advance the dynamics. However, in the CG simulations, a different approach was adopted. The rRESPA multi-timescale integrator^43^, which involves two integration levels, was implemented. For the calculation of non-bonded interactions, a timestep of 1 fs was employed, while a smaller timestep of 0.2 fs was chosen for the calculation of bonded interactions. The selection of a smaller timestep for the bonded interactions in the CG model was motivated by the presence of stiff bending and torsion potentials in the developed CG model. By using a smaller timestep, the MD runs remained stable, allowing for accurate and reliable simulations of the system's behavior. It's worth noting that despite using a smaller timestep for bonded interactions, the number of interactions that needed to be computed was significantly lower compared to what would be required in an all-atom system. This resulted in a considerably reduced computational cost. Additionally, it’s important to mention that the choice of timestep for simulations is inversely related to the lowest natural frequency of the simulated system. Therefore, the presence of stiff potentials necessitates a smaller timestep to accurately capture the system's dynamics. This choice in the CG simulation methodology ensures that the relevant dynamics and interactions within the system are accurately captured while maintaining computational stability.

In order to assess the mechanical behavior of the equilibrated systems, uniaxial stretching simulations were performed on both the all-atom and CG models of the silk. The constant strain minimization method was employed for this purpose, where the box dimension in the loading direction was incrementally increased by 0.005% of longitudinal strain. During each increment, energy minimization was performed while keeping the lateral dimensions fixed. The potential energy of the system was recorded at each step of the stretching process. Given that mechanical properties are closely linked to the potential energy of the system, we believe that a validation approach centered on the consistency of energy variation would be the suitable method in this context.

**Figure 8:**
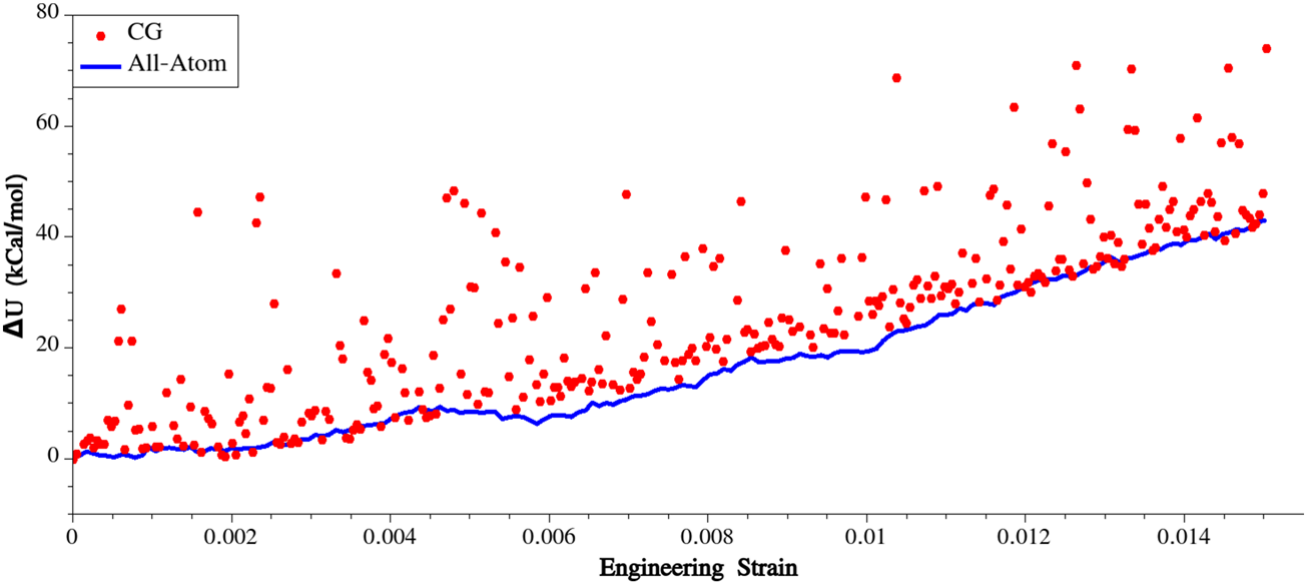
Variation of potential energy during uniaxial stretching.

The obtained variations in potential energy were compared between the all-atom and CG simulations, and the results are presented in Fig. 8. The plot demonstrates a relatively good agreement between the two variations in potential energy. However, it is important to note that the energy variation obtained from the CG simulation exhibits some noise. This noise can be attributed to the relatively low number of beads present in the simulation box, which in this case is 16 × 60 beads. The smaller system size (number of interacting sites) in the CG simulation compared to the all-atom simulation can lead to fluctuations and statistical noise in the calculated potential energy. Nonetheless, despite the noise, the overall trend and agreement between the energy variations obtained from both simulations indicate the effectiveness of the developed CG model in studying the mechanics of spider silk.

Thus far, we have confirmed the consistency of the CG simulation results with those obtained from all-atom simulations. This consistency implies that the CG model successfully captures the change in potential energy of the system under mechanical loading, aligning with the more detailed and computationally expensive all-atom simulations. The agreement between the two approaches provides confidence in the reliability and accuracy of the CG simulation method for studying the system under investigation. Validating the developed CG model, we’ll simulate spider silk under mechanical loading using various initial configurations. These configurations include fully ordered, semi-ordered, and fully random initial setups and the results are given in the following sections.

### Supplementary Information

Supplementary Information.

## Data Availability

The datasets used and analyzed during the current study are available from the corresponding author upon reasonable request.
