# Supplementary material for: Development of mechanically-consistent coarse-grained molecular dynamics model: case study of mechanics of spider silk
Source: Sci Rep. 2023 Nov 7;13:19316. doi: 10.1038/s41598-023-46376-6 (PMC10630411; doi:10.1038/s41598-023-46376-6)
Supplement: Supplementary file 1 — Supplementary Information. [file 41598_2023_46376_MOESM1_ESM.docx]

| 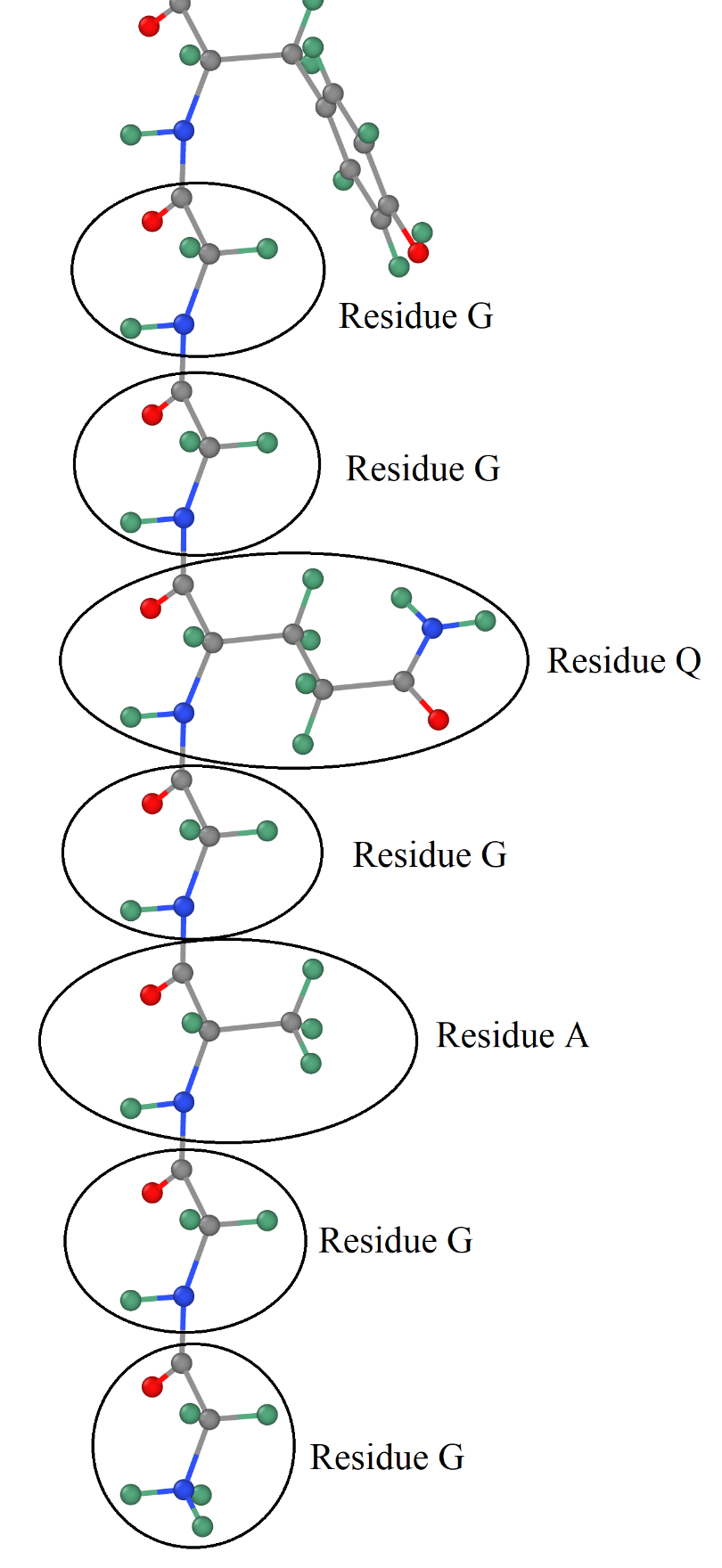 |
| --- |
| **Fig. S1.** One-bead-per-amino-acid residue approach used in development of the CG model |

| 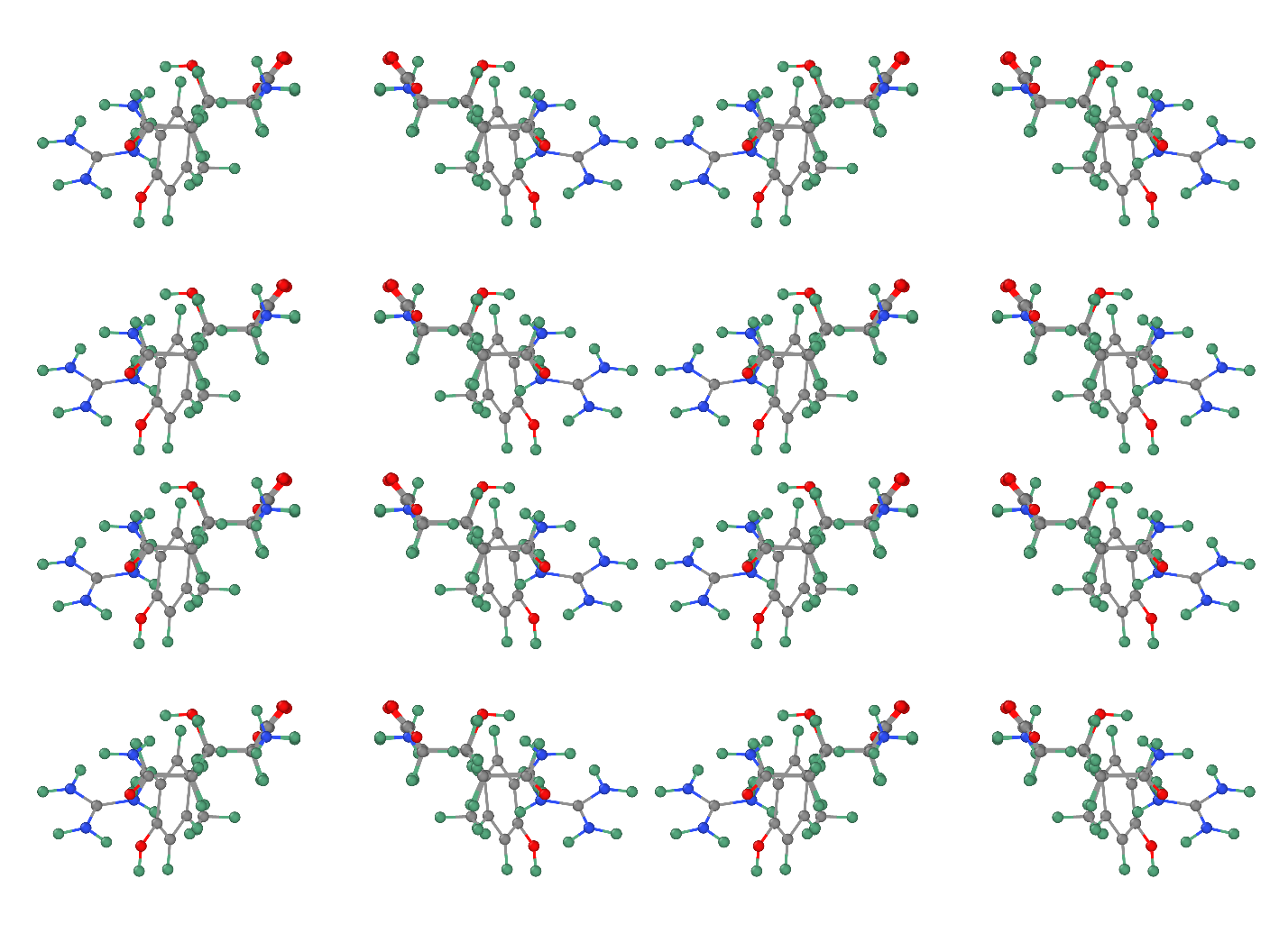 |
| --- |
| **Fig. S2.** Initial arrangement of chains in XY plane |

**Table S1. Calibrated parameters for stretching potentials:**

| Residue I | Residue J | K_b_ (kcal/mol/Å^2^) | Equilibrium bond length (Å) |
| --- | --- | --- | --- |
| 1 | 2 | 378.5418846964 | 3.1489779855 |
| 2 | 3 | 382.8500531535 | 3.2819539241 |
| 3 | 4 | 385.0734647010 | 3.2166031702 |
| 4 | 5 | 312.2708438882 | 3.9638715158 |
| 5 | 6 | 319.1365144190 | 4.2362446586 |
| 6 | 7 | 384.1545636464 | 3.1770786634 |
| 7 | 8 | 222.4372142364 | 3.1680376787 |
| 8 | 9 | 333.3069239096 | 5.3573571208 |
| 9 | 10 | 382.2880446814 | 3.1719208101 |
| 10 | 11 | 338.0392020377 | 3.4293535669 |
| 11 | 12 | 349.3358663893 | 3.9676458369 |
| 12 | 13 | 382.2904467378 | 3.1571439596 |
| 13 | 14 | 274.9359450413 | 3.7879342932 |
| 14 | 15 | 357.9373397253 | 4.4880414338 |
| 15 | 16 | 384.2403223462 | 3.2589540960 |
| 16 | 17 | 388.7426130034 | 3.1992707645 |
| 17 | 18 | 306.6988556702 | 2.8589040528 |
| 18 | 19 | 366.6131976963 | 4.5801725642 |
| 19 | 20 | 381.1304926986 | 3.2018414512 |
| 20 | 21 | 333.9766744272 | 3.4976090423 |
| 21 | 22 | 345.1162301795 | 4.0290197569 |
| 22 | 23 | 381.3165997152 | 3.1762958581 |
| 23 | 24 | 304.8386907135 | 3.7256714676 |
| 24 | 25 | 322.9411931574 | 4.1617825602 |
| 25 | 26 | 385.5149393527 | 3.2116315693 |
| 26 | 27 | 393.3990227609 | 3.1942926998 |
| 27 | 28 | 384.3288444441 | 3.2355025622 |
| 28 | 29 | 387.7657838309 | 3.2198033925 |
| 29 | 30 | 385.3774046617 | 3.2204362069 |
| 30 | 31 | 402.7819791463 | 3.2928968184 |
| 31 | 32 | 404.2962719219 | 3.2579088778 |
| 32 | 33 | 406.5532835912 | 3.2406109419 |
| 33 | 34 | 394.7485700879 | 3.2129966820 |
| 34 | 35 | 386.2820092525 | 3.1281880374 |
| 35 | 36 | 381.6059444899 | 3.2565217124 |
| 36 | 37 | 378.3210760156 | 3.1987469257 |
| 37 | 38 | 315.0990800151 | 4.0690154022 |
| 38 | 39 | 325.3805212123 | 4.2694162469 |
| 39 | 40 | 384.0140356324 | 3.1539984516 |
| 40 | 41 | 229.6983701387 | 3.2121626617 |
| 41 | 42 | 337.9098586165 | 5.3843788236 |
| 42 | 43 | 382.5392115470 | 3.1467546779 |
| 43 | 44 | 339.1785015404 | 3.4408151931 |
| 44 | 45 | 349.8823742399 | 3.9692132260 |
| 45 | 46 | 382.3531945323 | 3.1589986762 |
| 46 | 47 | 278.1278772606 | 3.8259082088 |
| 47 | 48 | 357.1551819327 | 4.4503670385 |
| 48 | 49 | 383.0121933528 | 3.2640887529 |
| 49 | 50 | 386.9316771232 | 3.2204427838 |
| 50 | 51 | 352.2481147480 | 4.5405807906 |
| 51 | 52 | 330.6364802291 | 3.2827473492 |
| 52 | 53 | 392.4142750069 | 3.0849023353 |
| 53 | 54 | 338.8225301484 | 3.5427945817 |
| 54 | 55 | 340.2115099336 | 4.0073212529 |
| 55 | 56 | 381.8745966819 | 3.1810665491 |
| 56 | 57 | 316.3122666381 | 3.8889362861 |
| 57 | 58 | 311.4720215072 | 4.2055772923 |
| 58 | 59 | 382.4349061033 | 3.2662882375 |
| 59 | 60 | 388.1836163161 | 3.6519205861 |

**Table S2. Calibrated parameters for bending potentials:**

| Residue I | Residue J | Residue K | K_a_ (kCal/mol/rad^2^) | Equilibrium bending angle (degree) |
| --- | --- | --- | --- | --- |
| 1 | 2 | 3 | 450.468492 | 160.7814333 |
| 2 | 3 | 4 | 175.428992 | 142.3941126 |
| 3 | 4 | 5 | 367.469155 | 137.6799388 |
| 4 | 5 | 6 | 1793.93542 | 96.81286638 |
| 5 | 6 | 7 | 379.456459 | 133.2565068 |
| 6 | 7 | 8 | 303.395338 | 100.0526876 |
| 7 | 8 | 9 | 3073.82332 | 90.2414381 |
| 8 | 9 | 10 | 437.052888 | 139.1868735 |
| 9 | 10 | 11 | 294.982389 | 125.5539455 |
| 10 | 11 | 12 | 1282.19911 | 116.2207291 |
| 11 | 12 | 13 | 360.057582 | 120.9735848 |
| 12 | 13 | 14 | 526.811608 | 128.8583025 |
| 13 | 14 | 15 | 1117.64323 | 92.38830772 |
| 14 | 15 | 16 | 459.112278 | 149.453524 |
| 15 | 16 | 17 | 199.389971 | 149.3368583 |
| 16 | 17 | 18 | 152.02672 | 104.853341 |
| 17 | 18 | 19 | 1231.61633 | 112.7455599 |
| 18 | 19 | 20 | 182.02094 | 162.9830706 |
| 19 | 20 | 21 | 415.516297 | 145.386573 |
| 20 | 21 | 22 | 1252.78094 | 109.3189624 |
| 21 | 22 | 23 | 455.925321 | 145.0832136 |
| 22 | 23 | 24 | 241.209 | 122.9734469 |
| 23 | 24 | 25 | 2206.65459 | 107.4471816 |
| 24 | 25 | 26 | 304.109137 | 112.3127311 |
| 25 | 26 | 27 | 250.998321 | 169.7443637 |
| 26 | 27 | 28 | 265.068227 | 141.6974223 |
| 27 | 28 | 29 | 221.900205 | 163.7097003 |
| 28 | 29 | 30 | 238.817199 | 164.0241012 |
| 29 | 30 | 31 | 189.175928 | 160.0083356 |
| 30 | 31 | 32 | 184.107668 | 158.0636185 |
| 31 | 32 | 33 | 179.351848 | 166.3472545 |
| 32 | 33 | 34 | 222.266539 | 163.5859875 |
| 33 | 34 | 35 | 236.326687 | 143.8740386 |
| 34 | 35 | 36 | 212.591439 | 142.9242168 |
| 35 | 36 | 37 | 211.877211 | 159.987149 |
| 36 | 37 | 38 | 395.927613 | 147.6135785 |
| 37 | 38 | 39 | 1931.65516 | 94.10805482 |
| 38 | 39 | 40 | 359.849338 | 129.4258967 |
| 39 | 40 | 41 | 303.464891 | 102.6976948 |
| 40 | 41 | 42 | 2981.33777 | 87.65696239 |
| 41 | 42 | 43 | 442.596108 | 144.1256829 |
| 42 | 43 | 44 | 276.015851 | 120.9779545 |
| 43 | 44 | 45 | 1312.37127 | 116.1342297 |
| 44 | 45 | 46 | 366.290479 | 121.3728181 |
| 45 | 46 | 47 | 482.09056 | 126.8404113 |
| 46 | 47 | 48 | 1082.21019 | 93.66741763 |
| 47 | 48 | 49 | 387.28724 | 147.7409867 |
| 48 | 49 | 50 | 201.459614 | 150.2848359 |
| 49 | 50 | 51 | 193.436975 | 141.4880901 |
| 50 | 51 | 52 | 1547.39412 | 104.3197292 |
| 51 | 52 | 53 | 161.626687 | 99.60710281 |
| 52 | 53 | 54 | 341.351295 | 142.0660966 |
| 53 | 54 | 55 | 1078.59308 | 106.0263164 |
| 54 | 55 | 56 | 426.921422 | 142.8542455 |
| 55 | 56 | 57 | 276.311858 | 125.5792417 |
| 56 | 57 | 58 | 1831.75494 | 100.7621575 |
| 57 | 58 | 59 | 318.797426 | 129.2656385 |
| 58 | 59 | 60 | 180.040022 | 143.4898066 |

**Table S3. Calibrated parameters for torsion potentials:**

| Residue I | Residue J | Residue K | Residue L | K_d_ (kCal/mol/rad^2^) | Equilibrium torsion angle (degree) |
| --- | --- | --- | --- | --- | --- |
| 1 | 2 | 3 | 4 | 206.713203 | 98.3260251 |
| 2 | 3 | 4 | 5 | 38.161727 | -121.08006 |
| 3 | 4 | 5 | 6 | 1356.65507 | 123.421624 |
| 4 | 5 | 6 | 7 | 113.558435 | -147.93201 |
| 5 | 6 | 7 | 8 | 76.5422339 | -21.336616 |
| 6 | 7 | 8 | 9 | 1838.43125 | 164.303685 |
| 7 | 8 | 9 | 10 | 78.4141893 | -164.42531 |
| 8 | 9 | 10 | 11 | 86.4980553 | -41.089208 |
| 9 | 10 | 11 | 12 | 901.115077 | 166.002712 |
| 10 | 11 | 12 | 13 | 125.665626 | -157.79813 |
| 11 | 12 | 13 | 14 | 211.471591 | 137.639945 |
| 12 | 13 | 14 | 15 | 1251.05517 | 148.853429 |
| 13 | 14 | 15 | 16 | 44.542035 | -117.44894 |
| 14 | 15 | 16 | 17 | 189.052623 | 94.795649 |
| 15 | 16 | 17 | 18 | 197.707823 | 138.178592 |
| 16 | 17 | 18 | 19 | 1217.0811 | 145.969067 |
| 17 | 18 | 19 | 20 | 54.1384392 | -153.13436 |
| 18 | 19 | 20 | 21 | 24.3643678 | 86.7925233 |
| 19 | 20 | 21 | 22 | 898.701494 | 135.210014 |
| 20 | 21 | 22 | 23 | 64.8192599 | -150.54677 |
| 21 | 22 | 23 | 24 | 62.4440855 | -75.673577 |
| 22 | 23 | 24 | 25 | 999.374526 | 163.440889 |
| 23 | 24 | 25 | 26 | 221.956093 | 168.189169 |
| 24 | 25 | 26 | 27 | 61.3054838 | 153.254115 |
| 25 | 26 | 27 | 28 | 103.307415 | -152.21568 |
| 26 | 27 | 28 | 29 | 55.7186033 | 124.233916 |
| 27 | 28 | 29 | 30 | 44.3974629 | 22.6464764 |
| 28 | 29 | 30 | 31 | 80.9906403 | 52.8525817 |
| 29 | 30 | 31 | 32 | 105.645116 | 0.15951138 |
| 30 | 31 | 32 | 33 | 59.6613874 | -17.66116 |
| 31 | 32 | 33 | 34 | 75.6203586 | -3.4777752 |
| 32 | 33 | 34 | 35 | 99.6721482 | 178.25228 |
| 33 | 34 | 35 | 36 | 119.581804 | -0.4166639 |
| 34 | 35 | 36 | 37 | 84.1244404 | -176.46607 |
| 35 | 36 | 37 | 38 | 40.7383097 | -110.61545 |
| 36 | 37 | 38 | 39 | 1190.55191 | 118.559619 |
| 37 | 38 | 39 | 40 | 114.616607 | -178.5877 |
| 38 | 39 | 40 | 41 | 84.3568813 | -19.940538 |
| 39 | 40 | 41 | 42 | 1813.93316 | 167.625912 |
| 40 | 41 | 42 | 43 | 69.6494753 | -172.28049 |
| 41 | 42 | 43 | 44 | 121.188292 | -54.721478 |
| 42 | 43 | 44 | 45 | 859.445297 | 172.727433 |
| 43 | 44 | 45 | 46 | 118.314121 | -161.71866 |
| 44 | 45 | 46 | 47 | 256.243619 | 133.398988 |
| 45 | 46 | 47 | 48 | 1239.81261 | 154.907588 |
| 46 | 47 | 48 | 49 | 60.8788743 | -138.08572 |
| 47 | 48 | 49 | 50 | 155.420057 | 120.162095 |
| 48 | 49 | 50 | 51 | 56.3166046 | 177.63344 |
| 49 | 50 | 51 | 52 | 874.51005 | 143.815163 |
| 50 | 51 | 52 | 53 | 416.843974 | -163.4307 |
| 51 | 52 | 53 | 54 | 41.6675354 | 80.0538398 |
| 52 | 53 | 54 | 55 | 1064.76338 | 159.716645 |
| 53 | 54 | 55 | 56 | 71.9354846 | -145.26157 |
| 54 | 55 | 56 | 57 | 62.90455 | -37.646891 |
| 55 | 56 | 57 | 58 | 1349.0968 | 157.719938 |
| 56 | 57 | 58 | 59 | 159.527354 | -142.48142 |
| 57 | 58 | 59 | 60 | 195.07037 | 122.345549 |

**Table S4. Calibrated LJ parameters:**

| Residue I | Residue J | $\varepsilon$ (kCal/mol) | $\sigma$ (Å) |
| --- | --- | --- | --- |
| G | G | 2.95054647 | 4.22405243 |
| A | A | 3.78891539 | 4.26844569 |
| Q | Q | 6.08502302 | 4.31115393 |
| Y | Y | 4.84277865 | 5.45197017 |
| L | L | 1.92947394 | 4.52541321 |
| S | S | 5.96318299 | 4.1276562 |
| R | R | 3.12321903 | 4.4454633 |
